# Chronic inflammation, neutrophil activity, and autoreactivity splits long COVID

**DOI:** 10.1038/s41467-023-40012-7

**Published:** 2023-07-14

**Authors:** Matthew C. Woodruff, Kevin S. Bonham, Fabliha A. Anam, Tiffany A. Walker, Caterina E. Faliti, Yusho Ishii, Candice Y. Kaminski, Martin C. Ruunstrom, Kelly Rose Cooper, Alexander D. Truong, Adviteeya N. Dixit, Jenny E. Han, Richard P. Ramonell, Natalie S. Haddad, Mark E. Rudolph, Srilakshmi Yalavarthi, Viktoria Betin, Ted Natoli, Sherwin Navaz, Scott A. Jenks, Yu Zuo, Jason S. Knight, Arezou Khosroshahi, F. Eun-Hyung Lee, Ignacio Sanz

**Affiliations:** 1 Department of Medicine, Division of Rheumatology, Lowance Center for Human Immunology, Emory University, Atlanta, GA USA; 2 Emory Autoimmunity Center of Excellence, Emory University, Atlanta, GA USA; 3 Department of Biological Sciences, Wellesley College, Wellesley, MA USA; 4 Department of Medicine, Division of General Internal Medicine, Emory University, Atlanta, GA USA; 5 School of Medicine, Emory University, Atlanta, GA USA; 6 Department of Medicine, Division of Pulmonary, Allergy, Critical Care and Sleep Medicine, Emory University, Atlanta, GA USA; 7 Department of Medicine, Division of Pulmonary, Allergy and Critical Care Medicine, University of Pittsburgh, Pittsburgh, PA USA; 8 MicroB-plex, Atlanta, GA USA; 9 Exagen Inc., Vista, CA USA; 10 Division of Rheumatology, University of Michigan, Ann Arbor, MI USA; 11 ImmuneID Inc., Waltham, MA USA

**Keywords:** Systems analysis, Chronic inflammation, Autoimmunity, SARS-CoV-2

## Abstract

While immunologic correlates of COVID-19 have been widely reported, their associations with post-acute sequelae of COVID-19 (PASC) remain less clear. Due to the wide array of PASC presentations, understanding if specific disease features associate with discrete immune processes and therapeutic opportunities is important. Here we profile patients in the recovery phase of COVID-19 via proteomics screening and machine learning to find signatures of ongoing antiviral B cell development, immune-mediated fibrosis, and markers of cell death in PASC patients but not in controls with uncomplicated recovery. Plasma and immune cell profiling further allow the stratification of PASC into inflammatory and non-inflammatory types. Inflammatory PASC, identifiable through a refined set of 12 blood markers, displays evidence of ongoing neutrophil activity, B cell memory alterations, and building autoreactivity more than a year post COVID-19. Our work thus helps refine PASC categorization to aid in both therapeutic targeting and epidemiological investigation of PASC.

## Abstract (web summary)

Post-acute sequelae of COVID-19 (PASC) has heterogenous presentation and complex etiology. Here the authors profile peripheral blood of patients with PASC and analyze by machine-learning to identify immune and serology features that allow the stratification of PASC into inflammatory and non-inflammatory types for better diagnosis and therapy-planning.

## Introduction

The COVID-19 pandemic resulting from the emergence of the novel beta-coronavirus SARS-CoV-2 has been deeply immunologically investigated^1,2^, and is characterized by significant heterogeneity in disease manifestations^3^, clinical outcomes^4^, and recovery^5^. A particularly important aspect of those investigations has become an increased focus on patients that, despite resolution of many of the symptoms associated with acute viral infection, experience ongoing complications^6^. These post-acute sequelae of COVID-19 (PASC), commonly referred to as “long COVID”, range both in manifestation and severity from anosmia to fatigue to joint pain persisting months or even years following the acute phase of disease^7^. Although a continuum of disease has been clearly documented from the acute phase in patient cohorts^8^, the US Center for Disease Control and the World Health Organization recognize PASC diagnosis at 4 and 12 weeks after COVID-19 onset, respectively, to allow for acute-phase response recovery^9,10^. While significant effort has generated an expansive collection of immunologic associations across a spectrum of COVID-19 disease courses, their differential resolution and potential contribution in PASC remains less clear^1^.

Reliable immunotypes of severe/critical versus mild/moderate COVID-19 dependent on, or contributing to, a high-inflammation environment have been identified in acute disease^11^. In particular, integration of systems approaches to immune assessment have identified prominent roles for myeloid activation^12^, neutrophil activity^13,14^ and cytotoxic T-cell responses^15^ as common features of severe illness. A striking observation in these patients was the collapse of germinal centers (GCs) responsible for classical pathways of B-cell development in patients that had succumbed to the illness^16^ and the emergence of antibody-secreting cells via an alternative extrafollicular (EF) pathway^17^. This pathway, previously described in human autoimmune diseases such as lupus^17,18^, has been demonstrated to generate virally targeted but cross-reactive responses resulting in de novo autoreactivity^19^. In patients recovering from severe illness with symptoms consistent with PASC, these autoreactive responses were identifiable for months.

Similar to the acute phase of disease, recent work investigating the early phase of COVID-19 recovery has identified immunotypes of PASC that might be predicted by early inflammation signatures, although those studies were limited in their window post recovery and reported waning immunologic association over time^8^. Other studies identify correlates of PASC that appear to be broader, such as cortisol levels, but ongoing associations with dysregulated immunity and ultimate pathophysiological significance of those signatures remain less clear^20^. As ongoing inflammation beyond the acute phase of infection and ongoing autoreactive development and persistence would have strong implications in potential treatment modalities, an integrated assessment of various aspects of immune dysregulation is required.

To this end, this study combines broad serological screening, clinical testing, and B-cell response characterization with novel machine-learning methods to identify common features of PASC not observed in donors experiencing uncomplicated COVID-19 recovery. We further identify an inflammatory subclassification of PASC with distinct clinical correlates, building autoreactivity, and strong evidence of ongoing innate and adaptive immune activation and response. Taken together, this work identifies biological signatures of PASC with potential diagnostic and therapeutic potential and establishes a clear disease subtype that is both easily identifiable and highly relevant to ongoing investigations of immunomodulatory therapy as a treatment modality in PASC.

## Results

### PASC patients display hallmarks of systemic inflammation

To understand the immunologic features underpinning the complex symptomatology associated with PASC, 97 patients were recruited from COVID-19 recovery clinics in Atlanta, GA, USA to provide blood samples and deep clinical documentation. Enrollees had a mean age of 50 years (range 21–81), 71 (73%) were female, and the majority were African American (59%) (Table 1). Fifty-seven (59%) had mild acute COVID-19 with the remaining requiring hospitalization. At the time of sampling, patients were a mean of 140 days from COVID-19 onset, with the most common self-reported PASC symptoms, including dyspnea (69%), fatigue (64%), and brain fog (47%) (Table 1). Due to inconsistency in formal PASC diagnosis criteria provided by major health organizations for minimum COVID-19 recovery period^9,10^, alongside significant data suggesting that acute-phase disease may predict PASC manifestations^8^, patient samples were collected across a wide range of recovery time points (22–446 DPSO) to understand disease development and potential resolution. Patients who were suspected or diagnosed with rheumatic diseases prior to COVID-19 diagnosis were excluded from the cohort.

**Table 1:** Patient data table

Characteristics	PASC patients (*n* = 97)	Uncomplicated COVID recovery (*n* = 26)
**Sex**		
Female	71 (73%)	10 (50 %)
Male	26 (27%)	13 (50%)
**Age, mean (range)**	50 (21–81)	40 (24–70)
20–39 years	16 (17%)	15 (58%)
40–59 years	60 (63%)	8 (31%)
60–79 years	18 (19%)	3 (12%)
>80 years	1 (1%)	0 (0%)
**Race/ethnicity**		
African American/Black	57 (59%)	7 (27%)
White	27 (27%)	14 (54%)
Hispanic	7 (7%)	2 (8%)
Asian	2 (2%)	3 (12%)
Unknown	4 (4%)	0 (0%)
**Acute COVID-19 severity**		
Asymptomatic	0	0
Outpatient	57 (59%)	23 (88%)
Hospitalized	37 (39%)	3 (12%)
ICU-admitted	3 (3%)	0
**Collection DPSO. Mean (range)**	140 (22–446)	110 (18–304)
0–3 months	39 (41%)	13 (50%)
3–6 months	34 (36%)	8 (31%)
6–12 months	20 (21%)	5 (19%)
>12 months	2 (2%))	0 (0%)
**PASC symptoms (self-reported)**		
Dyspnea	65 (68%)	
Fatigue	61 (64%)	
Brain fog	45 (47%)	
Cough	31 (33%)	
Headache	29 (31%)	
Chest pain	23 (24%)	
Depression	20 (21%)	
Myalgias	19 (20%)	
Weakness	19 (20%)	
Anxiety	18 (19%)	
Anosmia/dysguesia	15 (16%)	
Arthralgias	14 (15%)	

**Fig. 1:**
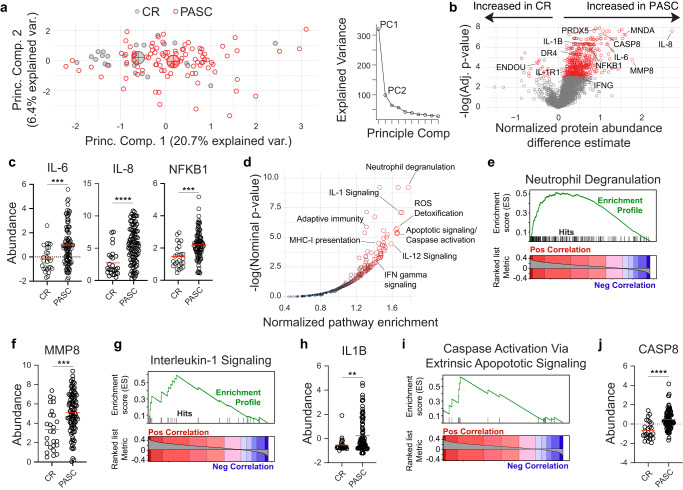
Inflammatory protein signatures in PASC. **a**–**j** Blood plasma from 97 PASC patients and 26 CR controls was assessed for 2925 independent protein features. **a** Left—Principal component analysis of PASC and CR cohorts. Large circles indicate population centroids. Right—Scree plot of the explained sample variance of the first ten principal components. **b** Feature-wise comparison between PASC and CR cohorts. Proteins of interest are labeled with significant differential abundances (Adj *P* < 0.001) highlighted in red. **c**, **f**, **h**, **j** Normalized abundance of indicated proteins with means displayed (red). **d** Reactome pathway analysis of proteins ranked by Spearman’s correlations with PASC diagnosis. Pathways of interest are labeled. **e**, **g**, **i** Gene set enrichment analysis of indicated pathways. **c**, **f**, **h**, **j** Two-way unpaired *T* test. *P < 0.05; **P < 0.01; ***P < 0.001, ****P < 0.0001. Source data are provided as a Source Data file.

Due to the critical role that systemic inflammation plays in COVID-19^21^, and early documented associations with PASC^21^, a high-dimensional screen of blood proteomics of almost 3000 independent targets was performed on patient plasma via the Olink Explore 3072 platform. A matched cohort of 26 donors with uncomplicated recoveries from COVID-19 at similar intervals post symptom onset were included as COVID-recovery (CR) controls (Table 1). Substantial heterogeneity in overall levels of blood markers was observed within the PASC group, with a large fraction of patients showing clear discrimination from the CR cohorts based on proteomic signatures, alone (Fig. 1a). More than 700 proteins displaying significantly increased abundance in the PASC cohort, with 20 additional proteins significantly decreased in comparison to CR controls (Fig. 1b). While elevated protein signatures were diverse in function, many of the most significant hits were inflammatory in nature and have been repeatedly identified as associates of the acute phase of severe COVID-19 including IL-6^22^, IL-8^23^, and NF-kB^24^ (Fig. 1b, c).

To identify broader trends in proteomic alterations within the PASC cohort we identified blocks of related proteins that were enriched in PASC subjects over CR controls (see “Software and analysis”). Although all biological pathways may not be evenly represented in the curated proteomics set, an analysis of pathway enrichment revealed several interesting biological pathways positively associated with the PASC cohort (Fig. 1d). Consistent with increased expression of IL-6 and IL-8 (Fig. 1c) neutrophil degranulation was the most enriched pathway in the set with matrix metalloprotease 8 (MMP8) and myeloid cell nuclear differentiation antigen (MNDA) highly increased (Fig. 1b, e, f). While multiple cytokine signaling pathways showed elevation in PASC, the IL-1B pathway was particularly responsive, with elevated levels of both the cytokine itself and the primary receptor elevated in the blood (Fig. 1b, g, h). The identification of IL-1R1, a transmembrane receptor, within the proteomics screen was reflective of an unanticipated ability of this method to identify proteins usually restricted to cellular compartments—potentially attributable to receptor cleavage via metalloprotease activity. Strong increased abundances of markers associated with cell death, including caspase 8 and the TNF death receptor, DR4, provide another potential explanation (Fig. 1b, j), suggesting that increased cellular debris from active cell death may be generally more abundant in these patients.

### ML identifies unanticipated features of PASC

**Fig. 2:**
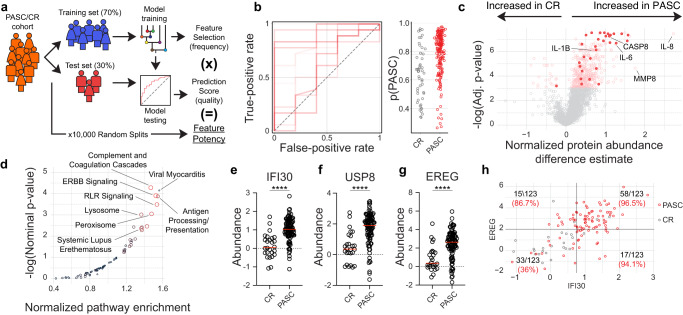
RF modeling identifies PASC features. **a**–**g** Blood plasma from 97 PASC patients and 26 CR controls was assessed for 2925 independent protein features. **a** Cartoon overview of random forest modeling approach and feature potency assessment. **b** Left—Receiver-operating characteristic (ROC) plot displaying ten models with randomized train/test splits classifying PASC and CR. Right—probabilistic classification plots for individual patients from the test sets derived from the ten models displayed in the ROC plot. **c** Feature-wise comparison between PASC and CR cohorts. Proteins of interest are labeled with the 30 features with highest feature potency highlighted. **d** KEGG pathway analysis of proteins ranked by feature potency in PASC discrimination modeling. **e**–**g** Normalized abundance of indicated proteins with means displayed (red). **h** Correlated but distinct information is provided by EREG and IFI30. Fraction of total samples and % purity indicated for each quadrant. **e**–**g** Two-tailed unpaired *T* test. *P < 0.05; **P < 0.01; ***P < 0.001, ****P < 0.0001. Source data are provided as a Source Data file.

Previous reporting on biological and clinical associations of PASC have yielded mixed results, with some prominent studies finding no clear biologic discrimination between patients with PASC and uncomplicated recovery^25^. Others identify clear distinctions at later time points in disease^20^. To take advantage of the high-dimensional nature of the proteomics dataset, we turned to Random Forests (RF), a class of supervised, nonparametric machine-learning (ML) models based on aggregating decision trees. RF models can be trained to take advantage of multiple independent or correlated features to generate probabilistic classifiers for a categorical response variable. (Fig. 2a). They are particularly well suited for this task, as blocks of co-regulated protein abundances within the dataset suggested that feature-wise parametric testing underlying feature significance testing may be underpowered (Supplementary Fig. 1). Further, trained RF models prioritize the ability of a feature to help distinguish between cohorts over measures of statistical deviation, thereby elevating the importance of features that may be less striking when considering only effect size and parametric significance but are critical discriminators between cohorts, nonetheless.

As RF model training is inherently random by design, potentially incorporating sub-optimal features for any individual model, we took a consensus modeling approach whereby 10,000 independent RF models were trained and evaluated on different data splits. This cross-validation approach is critical in ensuring that resulting models are not overfit to the dataset and maintain their generalizability to the broader patient population^26^. Despite clear proteomics signatures of PASC identified through traditional parametric testing (Fig. 1b), model performance was highly dependent on the patient cohort selected for inclusion within the training set (Fig. 2b) potentially indicating high heterogeneity within the PASC patient group. To identify individual features associated with strong model performance and PASC generalizability, proteins were individually scored for the following: (1) the frequency of incorporation into a final model over 10,000 iterations, (2) the importance of the feature for each model in group discrimination, and (3) the overall performance of the models it was integrated into (Supplementary Data 1). Perhaps unsurprisingly, the most influential features identified in this way were also significantly different between the PASC and CR groups through parametric testing, although they were not uniformly the most significant or differentially expressed features in the set (Fig. 2c).

Notably, many of the most significantly expressed inflammatory cytokines linked to neutrophil activity, including IL-6 and IL-8, were not identified among the top-scoring discriminators of PASC based on blood-based protein profiling (Supplementary Data 1). While neutrophil degranulation was highly represented in the overall differential expression analyses (Fig. 1d), it was conspicuously absent in an assessment of the biological pathways associated with high feature potency, which instead highlighted coagulation cascades, endothelial growth factor (EGF) signaling, antiviral sensing, and antigen presentation (Fig. 2d).

These pathways were reflected in the most potent individual discriminators of PASC within the feature set. IFI30, an interferon-gamma-induced mediator of peptide processing recently identified in the context of dysregulated neutrophil activity in COVID-19^13^, was incorporated as a key discriminator of PASC more than 96% of the time and associated with models with high predictive value (Fig. 2e and Supplementary Data 1). USP8, a component of T-cell antigen receptor (TCR) signalosome critical for thymocyte development, homeostasis, and proliferation^27^, was incorporated into models with similar frequency, although its selection was slightly less well associated with predictive power (Fig. 2f and Supplementary Data 1). Perhaps most interestingly, the epidermal growth factor (EGF), epiregulin (EREG), was consistently upregulated in PASC and was selected for incorporation into almost 90% of final predictive models (Fig. 2g and Supplementary Data 1). EREG has been identified as a critical mediator of IL-6/IL-17-induced upregulation of several EGF members and has been previously identified in COVID-19 as a correlate of inflammation^28^. It has also been suggested as a possible modulator of pain sensation in PASC^29^, and importantly, has been recently implicated in the immunologic maintenance of pulmonary fibrosis^30^. Together, features identified using this approach are extremely robust in the classification of PASC patients (Fig. 2h) and identify pathways of potential therapeutic value.

### Broad inflammation defines a subset of PASC

**Fig. 3:**
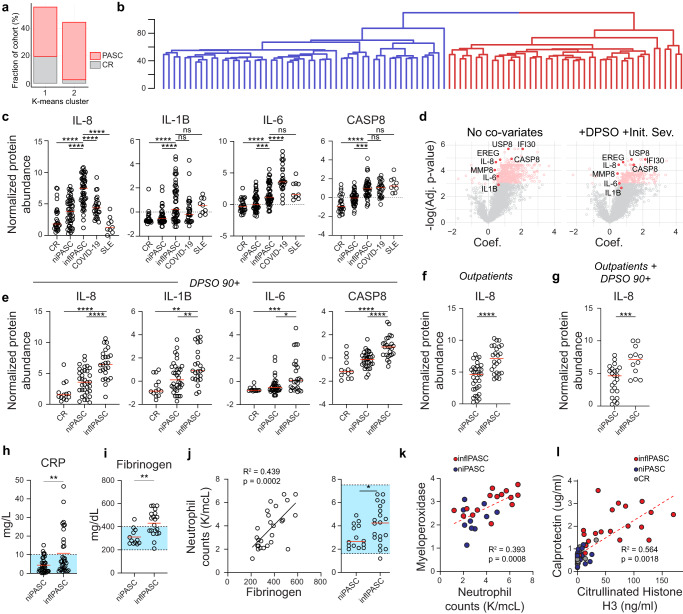
PASC subsets defined by inflammation. **a**–**e** Blood plasma from 97 PASC patients, 26 CR, 9 SLE, and 28 severe/critical COVID-19 controls was assessed for 2925 independent protein features. **a** Classification of PASC and CR patients following KNN clustering. **b** Hierarchical clustering of PASC patients. Major branches are differentially colored. **c** Quantification of indicated proteins in CR, niPASC, inflPASC, COVID-19, or SLE subjects with median displayed (red). **d** Generalized linear modeling of individual feature associations with PASC with either nocovariates assigned (left), or DPSO and IDS used as covariates (right). **e** Quantification of indicated proteins in CR, niPASC, or inflPASC patients more than 89 days post COVID-19 symptom onset with median displayed (red). **f** Quantification of IL-8 in niPASC and inflPASC patients with mild/moderate COVID-19. **g** Quantification of IL-8 in niPASC and inflPASC patients more than 89 days post mild/moderate COVID-19 symptom onset with median displayed (red). **h**–**j** Clinical blood testing of niPASC and inflPASC patient cohorts. Blue boxes indicate normal testing ranges. **h** C-reactive protein concentration in inflPASC and niPASC cohorts with mean displayed (red). **i** Fibrinogen in inflPASC and niPASC cohorts with mean displayed (red). **j** Neutrophil counts correlated with fibrinogen in PASC (left) and quantified between inflPASC and niPASC patients with mean displayed (red, right). **k** Neutrophil counts correlated with myeloperixidase expression in the plasma of PASC patients. **l** Calprotectin and Citrullinated Histone H3 concentration in the plasma of CR, niPASC, or inflPASC patients. **c**, **e** One-way ANOVA with multiple comparisons. **f**–**j** Two-tailed unpaired *T* testing. **c**–**j** *P < 0.05; **P < 0.01; ***P < 0.001, ****P < 0.0001. Source data are provided as a Source Data file.

Although RF-based approaches were promising in identifying PASC based on blood proteomics alone, expression of individual markers within the PASC cohort was highly heterogeneous. This was particularly true of protein sets associated with inflammation and neutrophil activity, and suggested that there may be subsets of the cohort with differential immunologic activity signatures (Fig. 1c, f, h). Consistent with this hypothesis, unsupervised clustering of the total recovery cohort into two subsets identified a clear subset of PASC patients clustering together with the CR cohort, while another set segregated almost entirely independently (Fig. 3a). Hierarchical clustering of the PASC cohort revealed a stark bifurcation of the overall cohort into two broad subsets (Fig. 3b). Assessment of the major markers of inflammation significantly upregulated in PASC such as IL-6, IL-8, and IL-1B all showed significantly increased abundances in one of the two PASC subsets, hereafter referred as the inflammatory PASC (inflPASC) subset (Fig. 3c, d). While non-inflammatory PASC patients (niPASC) showed elevated levels of some inflammatory cytokines, they often failed to reach significance in reference to the CR cohort (Fig. 3c). Of note, IL-8 and IL-1B signatures in the inflPASC cohort well-exceeded levels seen in severe/critical COVID-19 patients sampled in the acute phase of disease suggesting an inflammatory process unique to the recovery phase in this cohort.

The broad range of time points collected within the recovery cohort raised the possibility that that the stark separation of PASC patients based on proteomics assessment could simply be attributable to differences in recovery periods (DPSO) or initial disease severity (IDS) differences between the inflPASC and niPASC groups (Table 2). However, this was not the case. Generalized linear modeling of the proteomics data using DPSO and IDS as explicit covariates had little impact on the statistical significance of the markers as highly correlated with PASC (Fig. 3d). Similarly, filtering patients on those at more than 3 months post symptom onset, those with mild/moderate initial disease severities, or both, conclusively revealed this inflammatory state to be largely independent of either DPSO or IDS (Fig. 3e–g). This, combined with individual observations of inflPASC profiles more than a year post recovery, strongly suggest that these responses may be unexpectedly stable in a sizable proportion of patients.

**Table 2:** Subset patient data table

Characteristics	inflPASC (*n* = 49)	niPASC (*n* = 48)
**Sex**		
Female	34 (70%)	37 (77 %)
Male	15 (31%)	11 (23%)
**Age, mean (range)**	53 (32–77)	48 (24–81)
20–39 years	7 (14%)	11 (23%)
40–59 years	27 (55%)	32 (67%)
60–79 years	12 (24%)	5 (10%)
>80 years	0 (0%)	1 (2%)
**Race/ethnicity**		
African American/Black	34 (69%)	22 (46%)
White	9 (18%)	19 (40%)
Hispanic	5 (10%)	2 (4%)
Asian	1 (2%)	1 (2%)
Unknown	0 (0%)	4 (8%)
**Acute COVID-19 severity**		
Asymptomatic	0	0
Outpatient	24 (49%)	33 (69%)
Hospitalized	24 (49%)	14 (29%)
ICU-admitted	1 (2%)	1 (2%)
**Collection DPSO. Mean (range)**	123 (24–312)	156 (22–446)
0–3 months	24 (49%)	14 (29%)
3–6 months	16 (33%)	19 (40%)
6–12 months	9 (18%)	14 (29%)
>12 months	0 (0%)	1 (2%)

### Clinical distinctions in inflPASC

The identification of inflPASC based on clear differences in inflammatory signaling in the blood suggested that this heterogeneity may help explain the mixed results in clinically identifying PASC as a whole through standard clinical testing. This was confirmed, as broad markers of inflammation including C-reactive protein (CRP) could be readily identified in the plasma of inflPASC patients but not in niPASC counterparts (Fig. 3h). Fibrinogen was also elevated, with more than 50% of patients resulting a clinically abnormal test result (Fig. 3i). Although inside normal clinical ranges, neutrophil counts correlated tightly with fibrinogen levels in PASC patients and were significantly increased in the inflPASC cohort (Fig. 3j). Increases in neutrophil counts also correlated with proteins known to be released with neutrophil degranulation such as myeloperixidase (Fig. 3k). Testing for established biomarkers of neutrophil degranulation (calprotectin) and NETosis (citrullinated histone H3) revealed high levels of neutrophil activity observed exclusively in the majority of inflPASC patients tested. Altogether, these findings suggest that while available clinical tests fail to independently discriminate the inflPASC subgroup, they directly correlate with markers of neutrophil activity with strong established implications in neutrophil-based immunopathology^13,14^.

Although broad epidemiologic studies are necessary, clinical differences in disease presentation could also be observed between the niPASC and inflPASC cohorts (Supplementary Data 2). While many of the most prominent symptoms such as dyspnea and fatigue were present roughly equivalently between the groups, niPASC designation was associated with more than two-fold increased reporting of joint pain (23% vs. 6%), heart palpitations (14% vs. 6%), and anxiety (25% vs. 13%) compared to inflPASC. Muscle weakness was reported with increased frequency in inflPASC (13% vs. 33%). These differences in presentation became more prominent with time, with new discrepancies emerging between the groups with myalgia (17% vs. 29%) and numbness (7% vs. 13%) increased in inflPASC patients, alongside general weakness, at 90+ DPSO, thereby further confirming the persistence of this subset of patients well beyond the onset of disease. At more than 3 months following acute infection, almost 75% of niPASC patients reported brain fog in contrast to only 29% of inflPASC patients. Altogether, these data confirm a clinically distinct subset of PASC patients, independent of recovery time point, with differential inflammatory signaling, neutrophil activity, and clinical manifestations of disease.

### inflPASC patients show active B-cell profiles

**Fig. 4:**
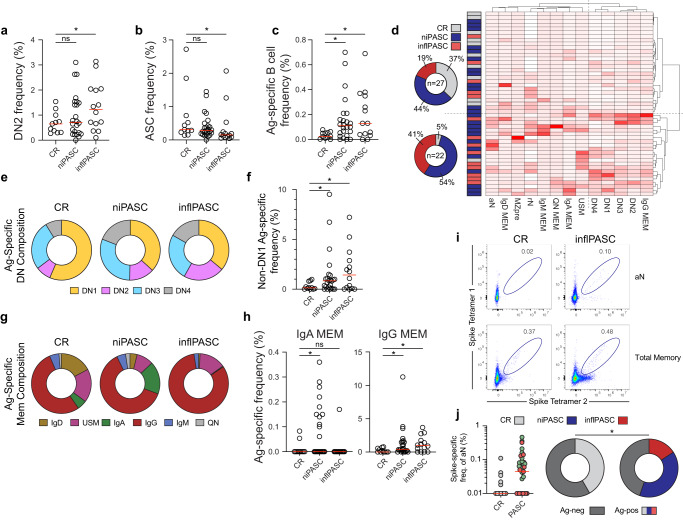
Altered B-cell responses in inflPASC patients. **a**–**j** Flow cytometric analysis B cells from 38 PASC and 11 CR patients. **a** DN2 B-cell frequency of B-cell-derived cells with median displayed (red). **b** ASC frequency of B-cell-derived cells with median displayed (red). **c** SARS-CoV-2 spike specific B-cell frequency of B-cell-derived cells with median displayed (red). **d** Heatmap of antigen-specific B-cell population frequency z-scores in CR (*n* = 11), inflPASC (*n* = 14) or niPASC patients (*n* = 24). Multivariate clustering of patients by Ward’s method is represented by dendrograms. **e** Ag-specific DN composition in CR, niPASC, or inflPASC patients. **f** Ag-specific frequency of non-DN1 (EF-associated) DNs in CR, niPASC, and inflPASC patients with median displayed (red). **g** Ag-specific memory composition in CR, niPASC, or inflPASC patients. **h** Ag-specific frequency of IgA or IgG memory in CR, niPASC, and inflPASC patients with median displayed (red). **i** Representative flow plots of CR (left) or inflPASC (right) ag-specific aN (top) or total memory (bottom) compartments. **j** (left) Proportion of donors in CR or PASC groups with observable ag-specificity in the aN compartment. Right—Ag-specific aN frequencies of CR or PASC groups from donors with an observable ag-specific aN compartment [from left]. **a**–**c**, **f**, **h** One-way ANOVA with multiple comparisons. *P < 0.05; **P < 0.01; ***P < 0.001, ****P < 0.0001. Source data are provided as a Source Data file.

To understand the nature of the cellular responses underlying the altered humoral targeting in the inflPASC group, antigen-specific flow cytometry was performed on 11 CR and 38 PASC patients (*n* = 14 inflPASC; *n* = 24 niPASC, Supplementary Fig. 2 and Supplementary Table 1). In the acute phase of severe COVID-19, naïve-derived extrafollicular B-cell responses correlated with the rapid expansion of antibody-secreting cells (ASCs)^17^, resulting in both antiviral and anti-self-reactivity^19^. Although mild in comparison to the acute phase of the disease, activity within the EF pathway was still observable through the elevation of DN2 B cells in PASC, with significant enrichment in the inflPASC subgroup. Interestingly, while strongly elevated ASCs were a hallmark of acute infection responses, PASC patients displayed frequencies on par, or even below CR donors (Fig. 4a, b).

Despite a muted ASC response, assessment of the antigen-specific B-cell compartment revealed increased ag-specific circulating B cells across both PASC subtypes (Fig. 4c). Using antigen-specific frequency of individual B-cell compartments to identify relevant repositories of SARS-CoV-2 specificity revealed a clear separation of patients. Almost all CR patients (10/11) were relegated to a cluster with a low frequency of ag-specific memory across most B-cell subsets (Fig. 4d) The other cluster, in contrast, was enriched for inflPASC patients (9/14) with increased ag-specific frequencies contained in the DN2, DN3, and IgG-class switched memory compartments (Fig. 4d). While CR patients showed ag-specific retention predominantly in the memory-associated DN1 compartment, inflPASC patients displayed increased spike reactivity in EF-associated DN populations (Fig. 4e, f). Similarly, while CR patients ag-specific memory compartment consisted of relatively balanced IgG and unswitched memory response, IgG responses dominated inflPASC memory retention at more than 80% of the overall population (Fig. 4g, h). Importantly, PASC patients were characterized by expansion of antigen-specific activated naïve (aN), B cells, suggestive of persistent viral triggering of de novo B responses. (Fig. 4j).

### inflPASC patients display altered humoral targeting

**Fig. 5:**
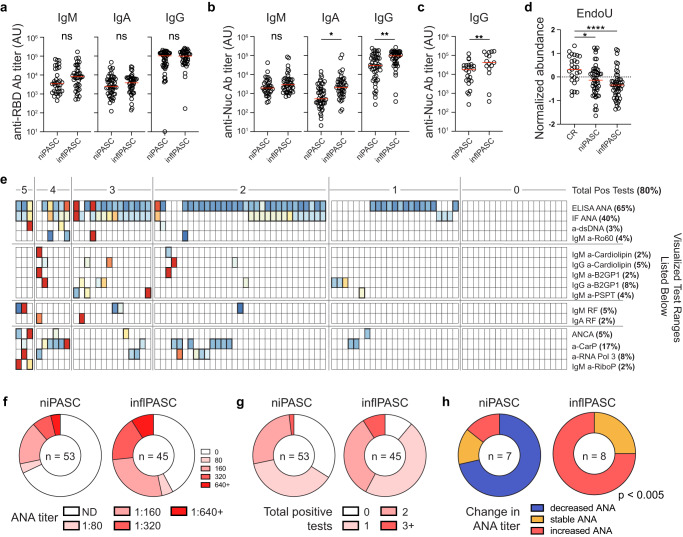
Autoreactive serology in inflPASC. **a**–**c** Plasma from 97 PASC patients was screened for reactivity against SARS-CoV-2. **a** Serological reactivity against the spike receptor binding domain in inflPASC and niPASC patients, by isotype, with means displayed (red). **b** Serological reactivity against nucleocapsid in inflPASC and niPASC patients, by isotype, with means displayed (red). **c** Serological anti-nucleocapsid responses in patients in inflPASC and niPASC cohorts more than 120 days DPSO with means displayed (red). **d** Normalized abundance of indicated proteins with means displayed (red). **e**–**g** Plasma from 96 PASC patients with mixed symptomology were screened by Exagen clinical laboratory for reactivity against 30 clinically relevant autoantigens. **e** Heatmap of patient results. Each column represents a single patient grouped by the total number of autoreactive positive tests that the patient displayed. Bolded boxes represent clinical positive tests with the color indicating the magnitude of the test result. Scale for each test is documented below the heatmap. **f** ANA titers in niPASC and inflPASC patients. **g** Total positive autoreactive tests in niPASC and inflPASC patients. **h** Trend in ANA titers in (*n* = 7) niPASC and (*n* = 8) inflPASC patients 1 year after initial plasma collection and screening. **a**–**c** Two-tailed unpaired *T* testing. **d** One-way ANOVA with multiple comparisons. *P < 0.05; **P < 0.01; ***P < 0.001, ****P < 0.0001. Source data are provided as a Source Data file.

In acute COVID-19, high levels of inflammation in critical illness drove higher levels of SARS-CoV-2-targeted antibody responses with significant cross-reactivity against self-antigens^19^. Serological testing of niPASC and inflPASC patients identified no clear serological difference between the groups in targeting the SARS-CoV-2 receptor binding domain binding, although IgM and IgA titers were slightly higher in the inflPASC group (Fig. 5a). By contrast, non-spike targeting was elevated in inflPASC. In particular, nucleocapsid antibodies were enriched in the inflPASC cohort across all isotypes tested, with significant increases in both IgA and IgG responses (Fig. 5b). As anti-nucleocapsid responses are known to diminish significantly over time^31^, it was again possible that the differences in targeting were attributable to the established trends in the inflPASC group towards earlier DPSO. However, restricting the analysis to patients collected more than 120 days post-diagnosis and eliminating the early time point bias of the inflPASC group showed similar enrichment of anti-nucleocapsid antibodies, suggesting that these differences in humoral immune targeting are stable over the time periods assessed (Fig. 5c). As previous studies have suggested that antiviral responses to unrelated viruses may be responsible for PASC manifestation, a screen of patient plasma antibodies against peptide libraries of more than 450 characterized human pathogens was performed. Although PASC patients showed a trend toward increased viral reactivity in general, no specific viral targets beyond SARS-CoV-2 could be identified as correlated with PASC (Supplementary Fig. 3).

Previously, non-spike targeting in multisystem inflammatory syndrome in children (MIS-C) correlated with the production of self-targeted antibodies^32^. Ag-specific retention in the EF compartment (Fig. 4d–f) and reductions in Uridylate-Specific Endoribonuclease (EndoU, Fig. 5d), a positive regulator of peripheral tolerance enforcement, further suggested that inflPASC patients might also be enriched for autoreactive targeting. To this end, plasma samples were screened against 30 clinically relevant autoantigens associated with connective tissue disorders. As in acute COVID-19, patients with PASC were enriched for autoreactivity with more than 75% showing reactivity against at least one autoantigen (Fig. 5e). Also similar to COVID-19, anti-nuclear antibody (ANA) testing showed broad positivity, although much of the cohort displayed low titers (1:80–1:160) of questionable clinical relevance. However, more than a third of patients displayed autoreactivity against 2 or more autoantigens, with some patients resulting 5 total positive tests (Fig. 5e). As in COVID-19, anti-carbamylated protein responses were enriched with 17% of patients testing positive, alongside an unexpected enrichment in RNA polymerase 3 reactivity across the cohort.

While autoreactivity was enriched across the entire PASC cohort, it was further emphasized within the inflPASC subset. As a broad measure of broken tolerance, inflPASC patients displayed both higher incidence (>55%) and higher titers of ANAs. Increased ANA titers were reflective of broader autoreactivity within the group which contained a higher percentage of patients with positive tests to two or more independent self-antigens (Fig. 5f, g). Of great interest, anti-neutrophil cytoplasmic antibodies (ANCA) were restricted to the inflPASC group (4/44). Further, of the six patients resulting positive tests for anti-beta-2-glycoprotein 1 (B2GP1) antibodies, associated with clotting abnormalities in both anti-phospholipid syndrome and COVID-19, 5 segregated into the inflPASC subset.

Critically, a targeted follow-up of patients roughly 1 year after initial visit revealed resolving ANA reactivity in niPASC patients (5/7) in contrast to the building reactivity in inflPASC patients (6/8) (Fig. 5h). Of the eight inflPASC patients with follow-up testing, three were initially collected 90 + DPSO and all showed increasing titers demonstrating clear evidence of building autoreactivity beyond the acute phase of COVID-19. Further, one inflPASC patient had developed new reactivity against dsDNA, opening the possibility of antigen walk and chronic autoimmune development.

### Classifying inflPASC through ML

**Fig. 6:**
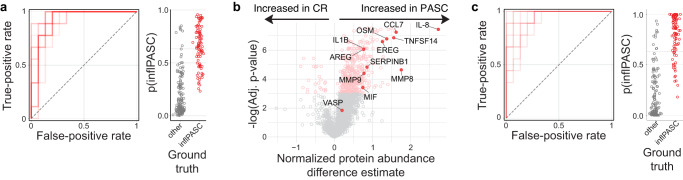
Identifying inflPASC in small feature sets. **a** Left—Receiver-operating characteristic (ROC) plot displaying ten models with randomized train/test splits classifying inflPASC from all other recoveries. Right—probability plots for individual “test” patients derived from the ten models displayed in the ROC plot. **b** Feature-wise comparison between PASC and CR cohorts. 12 manually curated proteins for reduced feature inputs are labeled. **c** Left—Receiver-operating characteristic (ROC) plot displaying ten models with randomized train/test splits classifying inflPASC from all other recovery using the manually curated list of 12 high-potency proteins identified in (**b**). Right—probability plots for individual “test” patients derived from the ten models displayed in the ROC plot.

The inflammatory milieu, neutrophilia, discordant self-reactivity and altered B-cell responses suggest that the inflPASC cohort may uniquely benefit from immunomodulation in the alleviation of disease burden. To accurately identify this specific patient subset, RF modeling was again implemented, this time classifying inflPASC patients from all other COVID-19 recovery. The resulting predictive modeling was extremely robust –10,000 models with randomized training/test set splits resulted in a mean ROC AUC of 0.95 (SD + /−0.04), suggesting that, unlike the generalized PASC cohort, inflPASC patients could be efficiently identified irrespective of the patient set selected for model training (Fig. 6a). This, combined with the broad set of proteins with increased abundance in inlfPASC patients strongly suggested that restricting our feature set to targets of known immunologic significance might still be effective in parsing the group. To this end, a list of 12 targets was manually curated from the most potent discriminators of inflPASC and used as inputs into a new RF model (Fig. 6b). Despite the restricted feature set, use of feature potency scores to guide parameter selection resulted in modeling that continued to be effective in discriminating the inflPASC group with a mean ROC AUC of 0.94 (SD + /−0.05), suggesting that full proteomics screening is not necessary to identify these patients (Fig. 6c).

## Discussion

Clinical heterogeneity in patients with PASC has made it challenging to identify clear biological associations with the disease^25^. Here, we suggest that PASC should be subclassified into (at least) two distinct conditions, characterized by the presence (inflPASC) or absence (niPASC) of broad inflammatory signatures consistent with high neutrophil activity and qualitative changes in B-cell memory and response. Using high-dimensional proteomics in combination with machine-learning-based modeling, we characterize clear signatures of generalized PASC strongly suggestive of dysregulation of discrete biologic processes underlying disease that may be tractable for both diagnostic and therapeutic purposes. Although traditional feature-wise testing showed an inflammatory component to PASC as a whole, a finding largely in agreement with emerging literature, pro-inflammatory cytokines such as IL-6, IL-8, and IL-1B were not identified as strong candidates for the discrimination of PASC when modeling the totality of blood protein content. Instead, signatures of complement and clotting cascades, active antigen processing, and EGFR signaling were more consistently associated across the group, with the identification of unanticipated targets, such as EREG, that may hold important diagnostic and therapeutic value. These findings are particularly interesting in light of recent work suggesting EREGs role, among other things, in pain sensation^29^, inflammatory dysregulation and autoimmunity^33^, and maintenance of pulmonary fibrosis^30^.

Likewise, proteomics-based clustering of PASC patients revealed a clear subset of patients closely associated with inflammatory immune signatures strongly suggestive of neutrophilic activity. Through readily available clinical testing, these inflPASC patients displayed neutrophil expansion correlating with both fibrinogen levels and biomarkers of degranulation and NET formation. Strikingly, many of the proteomics signatures associated with these patients, including IL-8, IL-1B, and IFI30, were highly reminiscent of recent work identifying transcriptional reprogramming of lung-infiltrating neutrophils that contributes to self-sustaining pathogenic neutrophilia in severe COVID-19^13^. This, alongside the known pathology associated with aberrantly regulated NET formation^34^ and the recent success of the neutrophil-modulator metformin in reducing PASC emergence by more than 40% in clinical trial cohorts^35^. It will be important to understand the significance of the correlation of these neutrophil signatures in these patients with clotting cascade members (Fig. 3i), their combined contribution to the clotting abnormalities identified in COVID-19 recovery and PASC^36^, and their detailed associations with clinical manifestations of PASC in larger longitudinal cohort studies. The streamlined approach to identifying these patients outlined here will greatly aid in those efforts.

It is important to note that while these clinical markers such as neutrophil counts and fibrinogen are elevated within the inflPASC group, they do not necessarily reflect ‘abnormal’ test results in all cases. That is, the testing of any marker independently may not, by itself, indicate clear disorder. Instead, the elevation of multiple markers, even when within “normal” ranges, seem to best reflect the broad inflammatory signals identified in the proteomics screen. This finding only emphasizes the need to develop tools capable of providing nuanced assessment across a variety of clinical parameters in patient classification. Similarly, it is important to acknowledge that niPASC is defined only as the absence of the robust inflammatory signature identified in inflPASC by comparison, and not as the absence of disease. As others have now shown^20^, and we show here (Fig. 2b, h), biological associations such as EREG upregulation and cortisol levels^8,20^ can be readily identified across a wide spectrum of PASC even independent of clear inflammatory signaling. It will be critical to understand how all of these signatures predict, and potentially contribute to, long-term patient morbidity.

A surprising finding from these data, in combination with the published literature^25^, is the difficulty in discriminating disease subtype through symptom presentation alone. While clear trends do emerge based on subclassification, symptom presentation alone is a poor discriminator of the inflPASC and niPASC groups despite their discordant underlying biology. It is notable, however, that in chronic autoimmune disorders, differences in underlying biology can heavily impact treatment success independently of overall disease presentation^37^. In the case of PASC, and based on the data presented here, two patients with highly similar symptomatic presentations might respond differently to immunomodulatory therapy. As a result, it is important to move beyond symptomatic presentation as a primary method for the classification of patients in therapeutic trial designs.

It is also important to understand how the signatures reported here might evolve over the course of the disease. While our cross-sectional approach defines clear lines between inflPASC and niPASC, and it is clear that inflPASC can present well beyond the expected phase of COVID-19 recovery, it is not yet clear if these presentations are mutually exclusive. In the case of reservoir-based viral reactivation as a main driver of PASC, as several have argued^38^, it could be that inflPASC manifestations are an observation of an inflammatory phase of cyclic reactivation rather than a discrete patient subtype. However, it is worth considering that the trending differences in symptomatology between the groups and differential persistence of autoantibodies argue against this interpretation. Alternatively, the distinction between inflPASC and niPASC could reflect a difference in the physiologic location of a viral repository. This would be consistent with the variability in memory isotype selection differences between the niPASC and inflPASC group despite the identification of antigen-specific aN B cells in both PASC subtypes strongly suggesting ongoing EF B-cell activation, presumably due to persistence viral antigens. If confirmed, the continued reliance on these EF-derived clonotypes for memory retention could have long-term implications in both ongoing cross-reactivity and self-targeting as well as the potential for self-sustaining autoimmune development in a subset of patients.

The overwhelming disease burden attributable to PASC worldwide^6^ demands that serious attention must be paid both to its accurate diagnosis as well as potential therapeutic avenues. The identification of a clear subclassification of PASC with a highly inflammatory presentation is an important first step. Based on these data, it is likely that these two PASC subclassifications may respond differently to the immunomodulatory therapies, particularly those targeted at neutrophil activity and autoimmune B-cell development, currently being investigated in large-scale clinical trials. Using machine-learning approaches, we have identified critical factors that can be used as positive classifiers of inlfPASC with a high degree of sensitivity and precision. While initial characterization of this heterogeneity required high-dimensional and unbiased screening, we found that a small subset of features that could be tested at scale, selected through novel assessments of feature potency, was nearly as performant when considered alone. Further, our integration of these data with classical in-clinic blood counts, clotting tests, autoreactive screening, and inflammatory marker assessment suggests that there may be several viable avenues to the positive identification of inflPASC patients without the need for highly specialized technology. These assessments could be easily integrated into ongoing clinical trials to understand if therapeutics exert discordant effects on specific patient groups and reduce the potential for false-negative outcomes due to patient heterogeneity.

## Methods

### Human subjects and clinical assessment

All research was approved by the Emory University Institutional Review Board (Emory IRB nos. IRB00058507, IRB00057983 and IRB00058271) and was performed in accordance with all relevant guidelines and regulations. Informed consent was obtained from all participants. Donors with uncomplicated COVID-19 recoveries (*n* = 26) were recruited using promotional materials approved by the Emory University Institutional Review Board.

Patients with PASC (*n* = 97) were referred by primary care providers or by self-referral to Emory University Midtown, Emory University Executive Park, and Grady Memorial Hospital PASC Clinics. Adults aged ≥18 years with documented SARS-CoV-2 antigen or anti-nucleocapsid antibody (64%), or those meeting the CDC COVID-19 clinical case definition who were experiencing new or worsening symptoms and were >14 days from COVID-19 onset (36%) were eligible. Sociodemographic, comorbidity, acute COVID-19, and PASC symptom data were collected by patient report through a review of systems and confirmed through medical record review. Clinical blood testing was performed on a subset of patients through routine care protocols.

Peripheral blood was collected in either heparin sodium tubes (PBMCs) or serum tubes (serum; both BD Diagnostic Systems). Study data were collected and managed using REDCap electronic data capture tools hosted at Emory University.

### Proteomic assessment and analysis

Frozen donor plasma was submitted for analysis using the commercially available Olink Explore 3072 platform based on previously published technological approaches^39^. Briefly, individual protein features are targeted by two independent antibodies carrying ssDNA tags. Upon dual-Ab binding, the ssDNA tags hybridize forming a PCR-competent substrate for amplification and sequencing. Protein abundances are normalized against in-plate and global controls and reported alongside sensitivity thresholds and quality control metrics. The resulting data was further assessed for quality with 1 PASC patient removed due to generalized protein abundances well outside of normal assay ranges. All samples were generally assessed for normal protein expression distributions and analyzed either through assessment tools provided by Olink in their custom “R” package, or through customized analysis pipelines developed in-house.

### COVID-19 multiplex immunoassay

SARS-CoV-2 antigens were coupled to MagPlex Microspheres of spectrally distinct regions via carbodiimide coupling and tested against patient samples as previously described^31^. Results were analyzed on a Luminex FLEXMAP 3D instrument running xPonent 4.3 software. Median fluorescent intensity (MFI) using combined or individual PE-conjugated detection antibodies (anti-IgA/anti-IgG/anti-IgM) was measured using the Luminex xPONENT software on the Enhanced PMT setting. The background value of the assay buffer was subtracted from the serum/plasma to obtain MFI minus background (net MFI). Serum and plasma samples were tested at 1:500 dilution.

### Quantification of neutrophil activity biomarkers

Cit-H3 levels in the plasma were quantified using the Citrullinated Histone H3 ELISA Kit (Cayman, 501620) according to the manufacturer’s instructions. Patient plasma was diluted 1:10 prior to assay loading. Calprotectin levels in the plasma were measured with the Human S100A8/S100A9 Heterodimer DuoSet ELISA (R&D Systems, DY8226-05) as per the manufacturer’s instructions. Patient plasma was diluted 1:500 prior to assay loading.

### Flow cytometry

Isolated PBMCs (10 × 10^6^) were centrifuged and resuspended in 75 μl FACS buffer (PBS + 2% FBS) and 5 μl Fc receptor block (BioLegend, no. 422302) for 5 min at room temperature. For samples stained with anti-IgG, it was observed that Fc block inappropriately interfered with staining, so a preincubation step of the anti-IgG alone for 5 min at 22 °C was added before the addition of the block. Next, 25 μl of antibody cocktail (Supplementary Table 1) was added (100 μl staining reaction), and samples were incubated for 20 min at 4 °C. Cells were washed in PBS, and resuspended in a PBS dilution of Zombie NIR fixable viability dye (BioLegend, no. 423106). Cells were washed and fixed at 0.8% paraformaldehyde (PFA) for 10 min at 22 °C in the dark before a final wash and resuspension for analysis.

Cells were analyzed on a Cytek Aurora flow cytometer using Cytek SpectroFlo software. Up to 3 × 10^6^ cells were analyzed using FlowJo v10 (Treestar).

### Autoreactivity screening

For autoimmune biomarker analysis, frozen plasma was shipped on dry ice to Exagen, Inc. (Vista, California, USA) which has a clinical laboratory accredited by the College of American Pathologists (CAP) and certified under the Clinical Laboratory Improvement Amendments (CLIA). Thawed plasma was aliquoted and distributed for the following tests: anti-nuclear antibodies (ANA) were measured using enzyme-linked immunosorbent assays (ELISA) (QUANTA Lite; Inova Diagnostics) and indirect immunofluorescence (IFA) (NOVA Lite; Inova Diagnostics); anti-double-stranded DNA (dsDNA) antibodies were also measured by ELISA and were confirmed by IFA with Crithidia luciliae; extractable nuclear antigen autoantibodies (anti-Sm, anti-SS-B/La IgG, anti-Scl-70 IgG, anti-U1RNP IgG, anti-RNP70 IgG, anti-CENP IgG, anti-Jo-1 IgG, and anti-CCP IgG) as well as Rheumatoid Factor (RF) IgA and IgM were measured using the EliA test on the Phadia 250 platform (ThermoFisher Scientific); IgG, IgM, and IgA isotypes of anti-cardiolipin and anti-β2-glycoprotein, as well as anti-Ro52, anti-Ro60, anti-GBM, anti-PR3, and anti-MPO were measured using a chemiluminescence immunoassay (BIO-FLASH; Inova Diagnostics); anti-CarP, anti-RNA-pol-III, and the IgG and IgM isotypes of anti-PS/PT were measured by ELISA (QUANTA Lite; Inova Diagnostics), while C- and P-ANCA were measured by IFA (NOVA Lite; Inova Diagnostics). All assays were performed following the manufacturer’s instructions.

### Phage immunoprecipitation sequencing and analysis

Frozen plasma samples were shipped to ImmuneID for analysis through their commercially available VirScan analysis pipeline based on previously published technological approaches^40^. Briefly, a custom T7 bacteriophage library consisting of 149,259 peptides tiling all protein-coding sequences from viruses with human hosts was constructed. Viral protein sequences were downloaded from Uniprot, collapsed on 90% identity, and bioinformatically parsed into 90 amino acid peptide tiles with 45 amino acid overlaps between adjacent tiles.

T7 bacteriophage libraries were aliquoted into 96-well plates and incubated with 20 μl each of protein A and G Dynabeads on a rotator for 4 h at room temperature. Next, plates were placed on a magnet, and supernatants were transferred to a fresh 96-well plate, to which we added patient plasma containing 2 μg of total IgG, and continued with the immunoprecipitation and washing steps, as previously described. Following the washes, protein A and protein G Dynabeads were resuspended in PCR master mix, amplified with 16 rounds of PCR, SPRI cleaned to remove primers, and indexed for sequencing with 8 rounds of PCR with primers containing Illumina p5 and p7 barcodes. NGS libraries were quantified on a Tapestation4200 and normalized for sequencing on an Illumina Nextseq2000 instrument. Each library received a minimum of 3 M reads.

PhIP-seq single-end DNA sequences were aligned to a library of 149,259 75 bp reference DNA sequences with the bowtie2 aligner (v2.0) using end-to-end matching. Read counts were summarized using samtools (v1.14) and collated into a counts matrix. The raw counts were converted to counts per million (CPM) using the “cpm” function from the R package edgeR (v3.36.0). CPM values for healthy controls were summarized by computing the peptide-wise mean and standard deviation across all healthy control samples. CPM values for each patient sample were collapsed by computing the peptide-wise minimum across technical replicates. Peptide-wise z-scores were then computed as:

$${Z}_{i,\,j}=({C}_{i,\,j}{{{{{\rm{-}}}}}}{\mu }_{j})/{{{{{{\rm{\sigma }}}}}}}_{j}$$

where *Z*_i,j_ is the z-score for patient *i*, peptide *j*; C_*i,j*_ is the minimum CPM for patient i, peptide j; *μ*_*j*_ is the mean of peptide j in the healthy control samples, and *σ*_*j*_ is the standard deviation of peptide j in the healthy control samples. For each patient, hits were identified as those peptides with *C*_*i*, *j*_ ≥ 10 AND *Z*_*i*, *j*_ ≥ 10.

### Software and analysis

Computational analysis was carried out in R (v3.6.2; release 12 Dec 2019). Heat maps were generated using the “pheatmap” library (v1.0.12), with data pre-normalized (log-transformed z-scores calculated per feature) before plotting. Clustering was carried out using Ward’s method. Custom plotting, such as biological pathway analysis, was performed using the “ggplot2” library for base analysis, and then post-processed in Adobe Illustrator. UMAP coordinates were generated using the ‘UMAP’ library, and then visualized through the “ggplot2” library package. GSEA analyses were performed using the GSEA desktop application using Reactome or KEGG gene sets. Statistical analyses were performed directly in R, or in GraphPad Prism (v8.2.1).

### Patient classification through machine learning

Random forest models were trained using “MLJ.jl” and “DecisionTrees.jl”. Hyperparameter tuning (maximum splits, minimum number of samples to allow split, minimum number of samples per leaf) for each class of models (CR vs PASC, inflPASC vs. Other) was performed independently using a subset of 80% of samples. Iterative training was performed as follows:

1. A stable random number generator seed was selected.
2. Samples were randomly assigned to training (80%) and test (20%) sets.
3. The model was trained on the training set using 1000 trees, and hyperparameters identified from the tuning step.
4. Gini (impurity) feature importance was calculated from training data.
5. AUC for the model was calculated based on classifications of the test set.
6. Importance scoring for feature $f$ and model $M$ was calculated as $Score(f|M) = Gini(f) * AUC(M)$.

### Reporting summary

Further information on research design is available in the Nature Portfolio Reporting Summary linked to this article.

## Supplementary information

Supplementary Information

Peer Review File

Description of Additional Supplementary Files

Supplementary Data 1

Supplementary Data 2

Reporting Summary

## Data Availability

The proteomics data have been deposited in Zenodo under accession number 8092298. All data are included in the Supplementary Information or available from the authors upon reasonable requests, as are unique reagents used in this Article.  Source data are provided with this paper.

## Source data

Source Data

## Code availability

The custom code used for this analysis has been deposited in Zenodo under accession number 8092298.
