# Supplementary material for: Chronic inflammation, neutrophil activity, and autoreactivity splits long COVID
Source: Nat Commun. 2023 Jul 14;14:4201. doi: 10.1038/s41467-023-40012-7 (PMC10349085; doi:10.1038/s41467-023-40012-7)
Supplement: Supplementary file 1 — Supplementary Information [file 41467_2023_40012_MOESM1_ESM.pdf]

Chronic inflammation, neutrophil activity, and autoreactivity splits Long COVID

First authors:
Matthew C. Woodruff (mcwoodr@emory.edu)
Kevin S. Bonham (kbonham@wellesley.edu)

Corresponding authors:
Matthew C. Woodruff (mcwoodr@emory.edu)
F. Eun-Hyung Lee (F.E.Lee@emory.edu)
Ignacio Sanz (Ignacio.sanz@emory.edu)

Supplemental Information

Supplementary Figure 1 – Correlation matrix of proteomics data

Supplementary Figure 2 – Flow cytometry B cell gating strategy

Supplementary Figure 3 – Viral peptidome reactivity in CR vs PASC

Supplementary Table 1 – Antibody lists for spectral flow cytometry

Supplementary figure 1

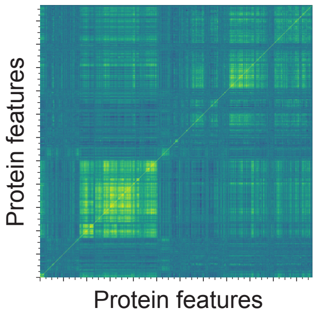

Supplementary Figure 1 – Correlation matrix of proteomics data
NxN matrix of total protein features representing large blocks of correlation structure in the protein feature dataset

Supplemetary Figure 2

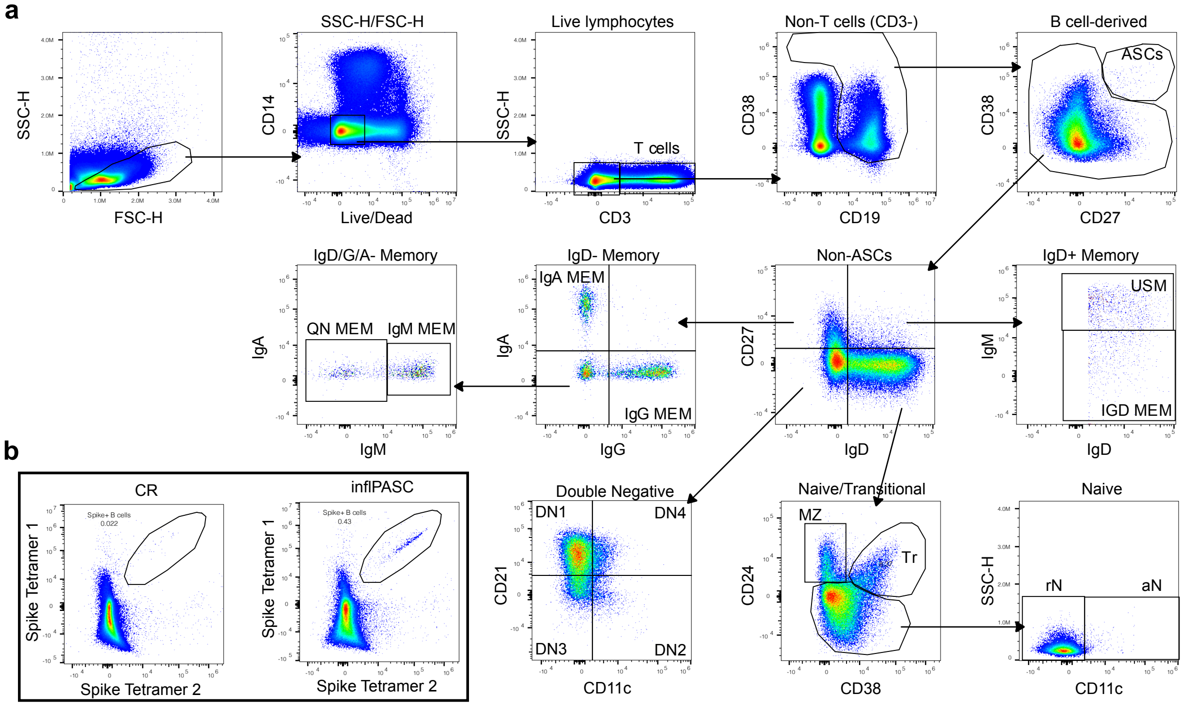

Supplementary Figure 2 – Flow cytometry B cell gating strategy
Flow cytometry gating strategy for B cell subpopulations identified in [Fig 4].

Supplementary Figure 3

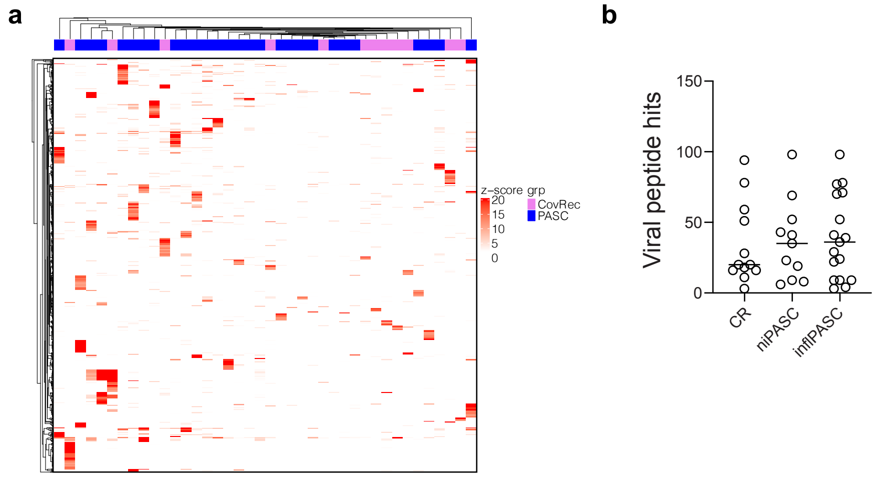

Supplementary Figure 3 – Viral peptidome reactivity in CR vs PASC
(a) Heatmap of reactivity against specific viral peptides across ~450 known human viral pathogens. PASC patients show slightly increased reactivity overall, but do not show common humoral targeting of any specific viral peptides (b) Overall anti-viral targeting of the viral peptidome in CR, niPASC, or inflPASC groups with medians displayed. All comparisons by One-way ANOVA are not significant.

Supplementary Table 1 – Antibody lists for spectral flow cytometry

antibody	conjugated	clone	company	Cat #	Dilution
CD14	BUV 805	M5E2	BD	612902	1:400
CD3	PE Fire640	SK7	Biolegend	344860	1:400
CD19	BUV 563	SJ25C1	BD	612916	1:400
CD20	BV 650	2H7	BD	563780	1:400
CD4	cFluorYG 584	SK3	Cytek	R7-20042	1:400
CD8	Spark Blue 550	SK1	Biolegend	344760	1:400
CD69	BUV737	FN50	BD	612818	1:100
PD-1	BV 785	EH12.2H7	Biolegend	329930	1:150
CD45RA	BV 570	HI100	Biolegend	304132	1:200
CCR7	BUV496	2-L1-A	BD	749827	1:200
CD24	BV 605	ML5	BD	562788	1:300
CD38	APC Fire810	HIT2	Biolegend	303550	1:300
CD27	BV750	O323	Biolegend	302850	1:200
Cd11c	APC Fire750	3.9	Biolegend	301646	1:100
CD21	PE-Dazzle 594	Bu32	Biolegend	354922	1:300
CXCR5	BV750	RF8B2	BD	747111	1:300
CXCR3	BUV395	1C6/CXCR3	BD	565223	1:80
CCR6	BV 480	11A9	BD	566130	1:200
CD138	APC-R700	MI15	BD	566050	1:100
HLA-DR	BB 700	G46-6	BD	745782	1:500
CD25	PE Fire700	M-A251	Biolegend	356146	1:200
CD127	BV711	HIL-7R-M21	BD	563165	1:200
IgM	BV 510	MHM-88	Biolegend	314522	1:200
IgD	Pacific Blue	IA6-2	Biolegend	348224	1:200
IgG	PE Cy7	G18-145	BD	561298	1:100
IgA	FITC	Polyclonal	Southern Biotech	2052-02	1:200
Streptavidin	PerCP	n/a	Biolegend	405213	
Streptavidin	BV 421	n/a	Biolegend	405225	
Streptavidin	PE	n/a	Biolegend	405204	
Streptavidin	Alexa Fluor 647	n/a	Biolegend	405237	
Live/Dead	Zombie NIR	n/a	n/a	423106	1:500

Supplementary Table 1 – Antibody lists for spectral flow cytometry
A complete list of antibodies used for the spectral flow cytometry including clones, sources, catalogue numbers, and dilutions used.
