# Supplementary material for: Chronic inflammation, neutrophil activity, and autoreactivity splits long COVID
Source: Nat Commun. 2023 Jul 14;14:4201. doi: 10.1038/s41467-023-40012-7 (PMC10349085; doi:10.1038/s41467-023-40012-7)
Supplement: Supplementary file 3 — Description of Additional Supplementary Files [file 41467_2023_40012_MOESM3_ESM.pdf]

Description of Additional Supplementary Files

Supplementary Data 1: PASC vs uncomplicated recovery feature importance list
Feature importance (potency) scores for all data features based on 10,000 RF classifications between PASC and CR patient groups (See methods).

Supplementary Data 2: Symptom distribution by PASC subtype classification
PASC symptom presentations between niPASC and inflPASC groups. Subgroup analyses including only patients more than 90 DPSO (middle), or with mild/moderate initial illness (right) are provided. Symptoms with 2x changes between groups are represented in bold.
