# Supplementary material for: Chronic inflammation, neutrophil activity, and autoreactivity splits long COVID
Source: Nat Commun. 2023 Jul 14;14:4201. doi: 10.1038/s41467-023-40012-7 (PMC10349085; doi:10.1038/s41467-023-40012-7)
Supplement: Supplementary file 6 — Reporting Summary [file 41467_2023_40012_MOESM6_ESM.pdf]

nature portfolio

Corresponding author(s): Matthew Woodruff, PhD; F. Eun-Hyung Lee, MD; Ignacio Sanz, MD

Last updated by author(s): Jul 5, 2023

Reporting Summary

Nature Portfolio wishes to improve the reproducibility of the work that we publish. This form provides structure for consistency and transparency in reporting. For further information on Nature Portfolio policies, see our Editorial Policies and the Editorial Policy Checklist.

Statistics

For all statistical analyses, confirm that the following items are present in the figure legend, table legend, main text, or Methods section.

n/a	Confirmed	
☐	☒	The exact sample size (*n*) for each experimental group/condition, given as a discrete number and unit of measurement
☐	☒	A statement on whether measurements were taken from distinct samples or whether the same sample was measured repeatedly
☐	☒	The statistical test(s) used AND whether they are one- or two-sided<br>*Only common tests should be described solely by name; describe more complex techniques in the Methods section.*
☒	☐	A description of all covariates tested
☒	☐	A description of any assumptions or corrections, such as tests of normality and adjustment for multiple comparisons
☐	☒	A full description of the statistical parameters including central tendency (e.g. means) or other basic estimates (e.g. regression coefficient) AND variation (e.g. standard deviation) or associated estimates of uncertainty (e.g. confidence intervals)
☐	☒	For null hypothesis testing, the test statistic (e.g. *F*, *t*, *r*) with confidence intervals, effect sizes, degrees of freedom and *P* value noted<br>*Give P values as exact values whenever suitable.*
☒	☐	For Bayesian analysis, information on the choice of priors and Markov chain Monte Carlo settings
☒	☐	For hierarchical and complex designs, identification of the appropriate level for tests and full reporting of outcomes
☐	☒	Estimates of effect sizes (e.g. Cohen's *d*, Pearson's *r*), indicating how they were calculated

Our web collection on statistics for biologists contains articles on many of the points above.

Software and code

Policy information about availability of computer code

Data collection	Study data were collected and managed using REDCap electronic data capture tools hosted at Emory University. Peripheral blood was collected in either heparin sodium tubes (PBMCs) or serum tubes (serum; both BD Diagnostic Systems). Frozen donor plasma was submitted for analysis using the commercially available Olink Explore 3072 platform. Flow cytometry was performed on a Cytek Aurora flow cytometer using Cytek SpectroFlo software (V3.0). Up to 3×106 cells were analyzed using FlowJo v10 (Treestar).
Data analysis	Software and analysis<br>Computational analysis was carried out in R (v3.6.2; release 12 Dec 2019). Heat maps were generated using the 'pheatmap' library (v1.0.12), with data pre normalized (log-transformed z-scores calculated per feature) before plotting. Clustering was carried out using Ward's method. Custom plotting, such as biological pathway analysis, was performed using the 'ggplot2' library for base analysis, and then post-processed in Adobe Illustrator. UMAP coordinates were generated using the 'UMAP' library, and then visualized through the 'ggplot2' library package. GSEA analyses were performed using the GSEA desktop application using Reactome or KEGG gene sets. Statistical analyses were performed directly in R, or in GraphPad Prism (v8.2.1).<br><br>Patient classification through machine learning<br>Random forest models were trained using `MLJ.jl` and `DecisionTrees.jl`. Hyperparameter tuning (maximum splits, minimum number of samples to allow split, minimum number of samples per leaf) for each class of models (CR vs PASC, inflPASC vs Other) was performed independently using a subset of 80% of samples. Iterative training was performed as follows:<br><br>1. A stable random number generator seed was selected<br>2. Samples were randomly assigned to training (80%) and test (20%) sets

3. The model was trained on the training set using 1000 trees, and hyperparameters identified from tuning step
4. Gini (impurity) feature importance was calculated from training data
5. AUC for the model was calculated based on classifications of the test set.
6. Importance scoring for feature f and model M was calculated as $Score(f|M) = Gini(f) * AUC(M)$

For manuscripts utilizing custom algorithms or software that are central to the research but not yet described in published literature, software must be made available to editors and reviewers. We strongly encourage code deposition in a community repository (e.g. GitHub). See the Nature Portfolio guidelines for submitting code & software for further information.

Data

Policy information about availability of data

All manuscripts must include a data availability statement. This statement should provide the following information, where applicable:

- Accession codes, unique identifiers, or web links for publicly available datasets
- A description of any restrictions on data availability
- For clinical datasets or third party data, please ensure that the statement adheres to our policy

The proteomics data have been deposited in Zonodo under accession number 8092298 [https://zenodo.org/record/8092298]. All data are included in the Supplemental Information or available from the authors upon reasonable requests, as are unique reagents used in this Article. Source data are provided with this paper where appropriate.

Research involving human participants, their data, or biological material

Policy information about studies with human participants or human data. See also policy information about sex, gender (identity/presentation), and sexual orientation and race, ethnicity and racism.

Reporting on sex and gender	This study was performed inclusively of both males and females, and blinded to gender and sexual orientation. Cohort data are available in Table 1.
Reporting on race, ethnicity, or other socially relevant groupings	This study was performed on a demographic cohort reflective of the racial and ethnic diversity of Atlanta, GA. USA. Cohort data are available in Table 1.
Population characteristics	This study was performed on adults, aged 20-81. Cohort data are available in Table 1.
Recruitment	Participants were recruited from Emory healthcare clinics, or voluntary draw sites overseen by Emory IRB protocols
Ethics oversight	Emory University Institutional Research Board

Note that full information on the approval of the study protocol must also be provided in the manuscript.

Field-specific reporting

Please select the one below that is the best fit for your research. If you are not sure, read the appropriate sections before making your selection.

[x] Life sciences [ ] Behavioural & social sciences [ ] Ecological, evolutionary & environmental sciences

For a reference copy of the document with all sections, see nature.com/documents/nr-reporting-summary-flat.pdf

Life sciences study design

All studies must disclose on these points even when the disclosure is negative.

Sample size	No sample size calculation was performed. Similar studies have been carried out by our group previously and reached statistically significant conclusions.
Data exclusions	One patient sample was excluded due to proteomics data QC failure.
Replication	Replication could not be done on this cohort due to limitations in both patient availability and cost.
Randomization	Allocation was determined by patient disease characteristics.
Blinding	Blinding was not relevant to the study.

Reporting for specific materials, systems and methods

We require information from authors about some types of materials, experimental systems and methods used in many studies. Here, indicate whether each material, system or method listed is relevant to your study. If you are not sure if a list item applies to your research, read the appropriate section before selecting a response.

Materials & experimental systems

n/a	Involved in the study
☐	☒ Antibodies
☒	☐ Eukaryotic cell lines
☒	☐ Palaeontology and archaeology
☒	☐ Animals and other organisms
☒	☐ Clinical data
☒	☐ Dual use research of concern
☒	☐ Plants

Methods

n/a	Involved in the study
☒	☐ ChIP-seq
☐	☒ Flow cytometry
☒	☐ MRI-based neuroimaging

Antibodies

Antibodies used

Target; Fluorophore; Panel; Clone; Vendor; Cat#; Dilution
antibody conjugated clone company Cat # Dilution
CD14 BUV 805 M5E2 BD 612902 1:400
CD3 PE Fire640 SK7 Biolegend 344860 1:400
CD19 BUV 563 SJ25C1 BD 612916 1:400
CD20 BV 650 2H7 BD 563780 1:400
CD4 cFluorYG 584 SK3 Cytek R7-20042 1:400
CD8 Spark Blue 550 SK1 Biolegend 344760 1:400
CD69 BUV737 FN50 BD 612818 1:100
PD-1 BV 785 EH12.2H7 Biolegend 329930 1:150
CD45RA BV 570 HI100 Biolegend 304132 1:200
CCR7 BUV496 2-L1-A BD 749827 1:200
CD24 BV 605 ML5 BD 562788 1:300
CD38 APC Fire810 HIT2 Biolegend 303550 1:300
CD27 BV750 O323 Biolegend 302850 1:200
Cd11c APC Fire750 3.9 Biolegend 301646 1:100
CD21 PE-Dazzle 594 Bu32 Biolegend 354922 1:300
CXCR5 BV750 RF8B2 BD 747111 1:300
CXCR3 BUV395 1C6/CXCR3 BD 565223 1:80
CCR6 BV 480 11A9 BD 566130 1:200
CD138 APC-R700 MI15 BD 566050 1:100
HLA-DR BB 700 G46-6 BD 745782 1:500
CD25 PE Fire700 M-A251 Biolegend 356146 1:200
CD127 BV711 HIL-7R-M21 BD 563165 1:200
IgM BV 510 MHM-88 Biolegend 314522 1:200
IgD Pacific Blue IA6-2 Biolegend 348224 1:200
IgG PE Cy7 G18-145 BD 561298 1:100
IgA FITC Polyclonal Southern Biotech 2052-02 1:200
Streptavidin PerCP n/a Biolegend 405213
Streptavidin BV 421 n/a Biolegend 405225
Streptavidin PE n/a Biolegend 405204
Streptavidin Alexa Fluor 647 n/a Biolegend 405237
Live/Dead Zombie NIR n/a n/a 423106 1:500

Validation

All antibodies have been validated by the manufacturer for use in targeting human proteins as indicated above.

Flow Cytometry

Plots

Confirm that:

☒ The axis labels state the marker and fluorochrome used (e.g. CD4-FITC).

☒ The axis scales are clearly visible. Include numbers along axes only for bottom left plot of group (a 'group' is an analysis of identical markers).

☒ All plots are contour plots with outliers or pseudocolor plots.

☒ A numerical value for number of cells or percentage (with statistics) is provided.

Methodology

Sample preparation

PBMCs were isolated from whole blood through ficoll gradient separation and frozen down in aliquots for future use. 5 million cell aliquots were thawed, and then stained with the antibody/antigen cocktail as detailed above.

Instrument	Flow cytometry was performed on a Cytek Aurora flow cytometer.
Software	Cytek SpectroFlo software was used for signal unmixing/data collection (V3.0). Up to 3×106 cells were analyzed using FlowJo v10 (Treestar).
Cell population abundance	NA
Gating strategy	The complete gating strategy is provided in Supplemental Figure 2.

☒ Tick this box to confirm that a figure exemplifying the gating strategy is provided in the Supplementary Information.
